# An Up-to-Date Overview of the Complexity of Genotype-Phenotype Relationships in Myotonic Channelopathies

**DOI:** 10.3389/fneur.2019.01404

**Published:** 2020-01-17

**Authors:** Fernando Morales, Michael Pusch

**Affiliations:** ^1^Instituto de Investigaciones en Salud, Universidad de Costa, San José, Costa Rica; ^2^Istituto di Biofisica, CNR, Genova, Italy

**Keywords:** myotonia, channelopathies, clinical and genetic variability, clinical correlations, functional analyses

## Abstract

Myotonic disorders are inherited neuromuscular diseases divided into dystrophic myotonias and non-dystrophic myotonias (NDM). The latter is a group of dominant or recessive diseases caused by mutations in genes encoding ion channels that participate in the generation and control of the skeletal muscle action potential. Their altered function causes hyperexcitability of the muscle membrane, thereby triggering myotonia, the main sign in NDM. Mutations in the genes encoding voltage-gated Cl^−^ and Na^+^ channels (respectively, *CLCN1* and *SCN4A*) produce a wide spectrum of phenotypes, which differ in age of onset, affected muscles, severity of myotonia, degree of hypertrophy, and muscle weakness, disease progression, among others. More than 200 *CLCN1* and 65 *SCN4A* mutations have been identified and described, but just about half of them have been functionally characterized, an approach that is likely extremely helpful to contribute to improving the so-far rather poor clinical correlations present in NDM. The observed poor correlations may be due to: (1) the wide spectrum of symptoms and overlapping phenotypes present in both groups (Cl^−^ and Na^+^ myotonic channelopathies) and (2) both genes present high genotypic variability. On the one hand, several mutations cause a unique and reproducible phenotype in most patients. On the other hand, some mutations can have different inheritance pattern and clinical phenotypes in different families. Conversely, different mutations can be translated into very similar phenotypes. For these reasons, the genotype-phenotype relationships in myotonic channelopathies are considered complex. Although the molecular bases for the clinical variability present in myotonic channelopathies remain obscure, several hypotheses have been put forward to explain the variability, which include: (a) differential allelic expression; (b) trans-acting genetic modifiers; (c) epigenetic, hormonal, or environmental factors; and (d) dominance with low penetrance. Improvements in clinical tests, the recognition of the different phenotypes that result from particular mutations and the understanding of how a mutation affects the structure and function of the ion channel, together with genetic screening, is expected to improve clinical correlation in NDMs.

## Neuromuscular Diseases

Neuromuscular diseases are a clinically, genetically, and biochemically heterogeneous group of more than 80 different entities (https://www.mda.org/disease/list), some of which share clinical and dystrophic features (1, 2). The main tissue affected (some diseases are multisystemic) in these diseases is the skeletal muscle, which is the organ in charge of locomotion and other body movements, and contributes to metabolic energy in multicellular organisms. Malfunction of this organ due to structural, physiological, or biochemical changes, often caused by specific genetic mutations, can lead to progressive muscle weakness/wasting with detrimental health consequences (3). Clinical features, such as disease severity, progression, age of onset of symptoms, and prognosis are highly variable (2). Hereditary neuromuscular diseases have been classified into several groups depending on the group of muscles targeted by specific gene mutations. However, clinically different phenotypes have been found in various patients with different mutations in the same gene, and even in separate patients carrying identical mutations (4, 5). The respective genes encode structural proteins, enzymes, ion channels, some of them causing muscular dystrophies (1–3). Muscular dystrophies, which can be inherited as dominant or recessive diseases, include limb girdle muscular dystrophies, congenital muscular dystrophies, dystrophinopathies, facioscapulohumeral muscular dystrophy, Emery–Dreifuss muscular dystrophies, and myotonic dystrophies. This review will focus on a group of disease that have been classified within the group of myotonic conditions, more specifically, the myotonic channelopathies.

## Myotonic Diseases

Electrical properties of the skeletal muscle fiber membrane (in a wide variety of different organisms) are characterized by a high resting membrane permeability for Cl^−^ ions. These Cl^−^ ions are transported by Cl^−^ ion channels belonging to the CLC family, which includes Cl^−^ ion channels and Cl^−^/H^+^ exchangers that are found in all phyla from bacteria to mammals (6–8). The ClC-1 channel is specifically present in skeletal muscle where it accounts for the chloride conductance, G_Cl_, which amounts to ~80% of the resting membrane conductance in resting muscles (9–15). Due to this high conductance and because the equilibrium potential of Cl^−^ is close to the muscle resting membrane potential, ClC-1 conducts membrane currents that inhibit muscle excitability (6). The effective Cl^−^ homeostasis is central in the generation and propagation of the action potential in muscle fibers.

Indeed, it is believed that the prominent role of G_Cl_ in action potential repolarization and membrane potential stabilization is related to the large cell size and the peculiar t-tubular system of skeletal muscle. If repolarization were mediated exclusively by K^+^ channels (as in most neurons), the extracellular K^+^ concentration in the restricted t-tubular space would rise significantly, leading to depolarization and eventual inactivation of Na^+^ channels (15). In fact, the “myotonic runs” of repeated action potential firing are partially caused by K^+^ accumulation (15). Importantly, a still open question is whether ClC-1 channels are actually preferentially located in the t-tubular membrane or in the surface sarcolemma (16–19).

It is well-known that excitation-contraction coupling, triggered by the nerve-impulse induced muscle action potential, is orchestrated by multiple factors (mainly ion channels) that lead to the release of Ca^2+^ from the sarcoplasmic reticulum. However, changes in the electrical properties of an excitable muscle, that can occur by both acquired or inherited bases, are well-recognized causes of muscle malfunction in humans (20). Reduction in muscle excitability may be the major cause of muscle weakness leading to fatigue (21), while hyperexcitability may lead to a sustained bursts of discharges that cause involuntary after-contractions, a phenomenon known as myotonia, the classical and leading sign of several hereditary diseases of skeletal muscle (20, 22). Therefore, myotonia is a clinical sign of skeletal muscle that results from an increased excitability of the muscle fiber membrane such that a single nerve stimulus triggers a burst of repetitive action potentials causing a delay in the temporal course of muscle relaxation (23). In patients, this condition can be detected as both electrical and clinical myotonia (24, 25). Electrical myotonia is detected on electromyographic (EMG) tests as repetitive muscle fiber potential discharges, with waxing and waning frequency and amplitude with a firing rate between 20 and 80 Hz, while clinical myotonia is physically demonstrated by slowed muscle relaxation during repetitive hand grip, eye closure, or after tapping various muscles, such as the finger extensors (26).

Symptomatically, patients experience myotonia as stiffness at the beginning of motion, better exemplified during initial attempts at relaxation of hand grip (grip myotonia) and following percussion of the muscle located in the bases of the thumb (percussion myotonia) (27). Myotonia can ameliorate with repeated movements, a phenomenon known as warm-up, but also, it can get worse with activity and become paradoxical (28, 29).

Diseases associated with this sign are collectively termed myotonias and according to other clinical features (progressive muscle wasting and weakness, dystrophic changes), they are classified as: (1) dystrophic myotonias (DM) and (2) non-dystrophic myotonias (NDM). Myotonic dystrophy type 1 (DM1) and type 2 (DM2), caused by the expansion of unstable microsatellites, belong to the first group, and are beyond the scope of this review. A group of five diseases, collectively called myotonic channelopathies, caused by mutations in voltage-gated Na^+^ and Cl^−^ channel genes, belong to the second group (30). This review is focused on the latter group of diseases.

## Non-Dystrophic Myotonias

Dominantly or recessively inherited disorders caused by ion channel dysfunction include myotonia congenita (MC) (Thomsen's disease and Becker type myotonia), paramyotonia congenita (PC), hyperkalemic periodic paralysis (hyperPP) with myotonia and the sodium channel myotonias (SCM). These diseases show distinctive clinical features that allow separating them from the myotonic dystrophies. They are divided into two groups: the chloride and the sodium channelopathies. The first group, called myotonia congenita (MC), is subdivided in Thomsen's disease and the Becker generalized myotonia. The other group of three diseases listed above belong to the sodium channelopathies (24, 25, 30–32).

## Phenotype of Myotonic Channelopathies

### Chloride Channelopathies

For precise control of muscle contraction by nerve activity, in normal conditions, a single nerve action potential triggers only a single muscle action potential. This is to a large extent guaranteed by the large G_Cl_ that aids in repolarizing the action potential and, once repolarized, continues to stabilize the membrane potential, preventing thereby the insurgence of a train of action potentials (33). Reduction of the Cl^−^ conductance in the skeletal muscle causes myotonia congenita (MC), the most common ion channel disease (34). The first description of this pathology dates back to the late nineteenth century, when Danish physician Asmus Julius Thomas Thomsen described it for himself and for some of his family members, with an autosomal dominant inheritance pattern (35). The dominant form of MC was called Thomsen's disease (DMC) after that (36). Almost a century after that first description, German professor Peter Emil Becker described a MC variant with autosomal recessive inheritance pattern, a variant that was called recessive generalized myotonia or Becker myotonia (RMC) (37). MC is electrophysiologically characterized by presenting increased excitability of the muscular fiber, which is due to repetitive action potentials of the muscle membranes; this is reflected in clinical myotonia, muscular stiffness (that is worse after rest) and hypertrophy (38, 39). Myotonia in MC is clinically highly variable, ranging from myotonic discharges only detectable on the EMG test to disabling muscle stiffness at an early age (5). The leg muscles are commonly affected and handgrip myotonia is detected in about 3/4 of patients (22). Almost every skeletal muscle in the body might show muscular stiffness, but it is ameliorated by exercise (warm-up phenomenon). The clinical picture depends on whether the disease is inherited as autosomal dominant or autosomal recessive. RMC is more common (in most countries) and severe than DMC (5, 24, 25, 40–42). In DMC, age of onset is usually at birth or very early in infancy. The child might show unusually defined muscles in the extremities, delayed relaxation of the eyelids after forceful closure following sneezing or during crying, and hypertrophy is rare in childhood but common in adulthood (31, 43). Severity varies from mild to moderate, there is no progression of the symptoms, and patients can experience a normal life (43). In RMC, age of onset is usually later than in DMC (25, 43), but it has been also reported that age of onset could be earlier in RMC (44). Myotonia is generalized with moderate to pronounced hypertrophy, where many patients use to have a body-builder appearance due to hypertrophy triggered by the involuntary after-contractions (20, 43). Myotonia is more severe in the recessive variant and usually, typical transient muscular weakness (lasting from seconds to as long as 30 min) is also observed (10, 45), which may lead to recurrent falls (31, 34). Muscle strength is normal in this variant but the disease slowly progresses in some patients (34, 46).

### Sodium Channelopathies

The upstroke of the action potential is mediated by opening of voltage-gated Nav1.4 sodium channels that generate an inward Na^+^ current that renders the cells positive inside (depolarization) (31). Malfunction of these channels cause several human hereditary diseases of the skeletal muscle. Diseases belonging to this group are: paramyotonia congenita (PC), hyperkalemic periodic paralysis (HyperPP) with myotonia and the sodium channel myotonias (SCM). Similar to MC, these diseases are characterized by increased muscle membrane excitability that leads to repetitive action potentials; however, the underlying physiological defect is different compared with the chloride channelopathies (24, 47). Symptoms in sodium channelopathies are episodic and vary from patient to patient and also from time to time on each affected individual (47). Clinical myotonia, affecting hands, face, upper and lower extremities is generalized, going from absent to very severe, depending on the disease, and usually gets worse with exercise; while electrical myotonia may be diffusely present in all muscle and in some cases, may worsen due to cold exposure, which may also induce weakness in some patients, and clinical myotonia. The length of the attack is very variable, lasting from minutes up to 7 days. Muscle hypertrophy is not as common as in the chloride channelopathies, but it is present in variable degrees in some patients. Age of onset of the disease is usually in the first decade and it might progress later in life in some patients (25, 27, 48–50).

PC was first described by Eulenburg (51). Age of onset of first symptoms is at birth or during the first decade of live, affecting mainly the muscles from neck, face and upper limbs. As indicated by its name, PC shows paradoxical myotonia, which gets worse with repeated movements and/or cold exposure. PC show variable weakness, which becomes evident after myotonic stiffness attacks, the major symptom in PC (20, 47). PC patients may experience HyperPP weakness-like phenotype after pronounce cooling or vigorous exercise (20).

In HyperPP, age of onset of the first symptoms (recurrent episodes of weakness as the main symptom) is during childhood. Symptoms are triggered by ingestion of K^+^-rich food, rest after vigorous exercise, and other environmental factors (47, 52–54). The episodes of weakness vary from minutes to several hours, with normal weakness between attacks. Patients affected with HyperPP frequently have myotonia (symptomatically in 12.5% or by EMG in 50% of patients), which can be from mild to moderate, particularly with the onset of a weakness attack. Some patients develop paramyotonia, highlighting the extensive clinical overlapping between these two conditions (20, 47, 53, 55).

SCM is less well-defined with onset of first symptoms from childhood, as in acetazolamide-responsive myotonia, to adolescence, as in myotonia fluctuans (47). Myotonia can be present from mild (in myotonia fluctuans) to severe (in myotonia permanens affecting swallowing and breathing); no periodic paralysis has been observed in SCM patients, while there are some patients that have shown potassium-aggravated myotonia (K^+^-sensitive myotonia) (20).

## Genetics and Mutation in Myotonic Channelopathies

### Chloride Channelopathies

As mentioned above, MC is the most common hereditary ion channel disorder in humans, showing a prevalence between 1:23,000 and 1:50,000 for the recessive form (Becker Myotonia), while the dominant form (Thomsen's disease) is a bit less common in most countries (40, 42, 56). Both conditions are caused by mutations in the chloride voltage-gated channel 1 (*CLCN1*) gene (10, 57–59), which is located in chromosome 7q35 and encodes the voltage-gate chloride channel (ClC-1), belonging to the CLC family of chloride channels (60, 61). Although the pathogenesis of MC is not fully understood, it is well-known that mutations in *CLCN1* produce a reduction of the Cl^−^ conductance that leads to membrane hyperexcitability, triggering repetitive action potentials (24, 25, 31). The channel conducts chloride ions over the entire physiological voltage ranges and is the major mediator of chloride conductance in skeletal muscle (13, 14, 31, 62, 63). Two subunits of the channel are required to come together to form the functional channel, and thus, work as double-barreled homodimers (64–66). The *CLCN1* gene has 23 exons, with more than 200 different mutations described in this disease (4, 41, 43, 67, 68) (http://www.hgmd.cf.ac.uk/ac/index.php). Mutations are found through the entire gene sequence, being present in the N-terminal, transmembrane, and C-terminal domains of ClC-1. Different types of mutations have been found in the *CLCN1* gene, including nonsense, splice-site, missense, frameshift (insertion/deletions), and deletion/duplication mutations, with exon eight becoming a hot spot for DMC (20, 41, 67, 69–71). The recessive inheritance is conceptually explained by a loss-of-function effect caused by the mutations without significantly impacting on the formation or function of dimeric ClC-1 channels. On the other hand, the dominant inheritance is explained by a dominant-negative effect of mutated subunits on heteromeric mutant/WT channels. Most of the 200 different mutations identified and described behave as recessive, with the majority of the patients being compound heterozygous (carriers of two different recessive mutations). Only about 27 mutations have been associated with DMC, while about other 59 mutations have an unclear inheritance pattern, are sporadic or have been also shown to display a recessive inheritance pattern (http://www.hgmd.cf.ac.uk/ac/index.php). Therefore, a clear distinction between dominant and recessive mutations is not always possible (5, 39, 41, 43, 72–74). Thus, far, there is no other clinical phenotype associated with mutations in the *CLCN1* gene.

### Sodium Channelopathies

Na^+^ channelopathies are not as common as Cl^−^ channelopathies, showing a combined prevalence of about 1:100,000 (42). These disorders are caused by mutations in the sodium voltage-gated channel alpha subunit 4 (*SCN4A*) gene, which is located in chromosome 17q23 and encodes the voltage-gated sodium channel (Nav1.4) of skeletal muscle (75–77). They are a heterogeneous group of autosomal dominant disorders with high penetrance (20, 55). Mutations in *SCN4A* cause disruption of fast inactivation of the channel, which can be incomplete or slowed (78–80), leading to repetitive action potentials (myotonic runs) and consequent intracellular sodium accumulation that depolarizes muscle cells and can lead to inactivation of the Na^+^ channels (25, 31, 32, 47). Depending if depolarization is mild or not, myotonia or paralysis might appear, respectively (81). Nav1.4 is a channel formed by a single unit of Nav1.4 protein, which contains four repeated domains (DI-DIV), each one consisting of six transmembrane segments (S1–S6). The loops between S5–S6 segments from the four domains come together to form the ion-conducting pore, acting as a selective filter. Meanwhile, the S4 segment of each domain is in charge of sensing the voltage changes (31, 32, 47). The *SCN4A* gene has 24 exons, with about 83 different mutations described in the gene, but only about 65 of them have been associated with myotonia (40, 82–86) (http://www.hgmd.cf.ac.uk/ac/index.php). All *SCN4A* mutations correspond to missense mutations, with the single exception of a deletion/insertion mutation located in the splice site in intron 21 (87, 88). All *SCN4A* myotonia mutations studied produce a gain-of-function effect of Nav1.4, resulting in defects of channel inactivation or enhancement of activation, which explain the dominant inheritance pattern of the diseases (20, 82). Mutations have been located through the entire gene sequence, but depending on the disease, they tend to group differentially. For instance, mutations associated with HyperPP with myotonia are generally located in the inner regions of the transmembrane segments or in the intracellular interlinking loops in repeat domains DII and DIV of the channel, eliciting a persisting inward sodium current, which impairs repolarization and increases membrane excitability (82, 89). In PC, mutations have been found throughout the gene, with exon 24 appearing to be a hot spot (90), but their impairment of fast inactivation is less notorious than the one associated with HyperPP (81). Regarding SCM, although mutations have been found throughout the gene, there are more likely located in the N terminus of the channel, in repeat domain D1, particularly in the inactivation gate (82). Interestingly, in recent papers, the authors describe several *SCN4A* mutations, previously reported in unrelated myotonia-positive families, new or *de novo* mutations, that contribute to apnea during the physiological stress of seizures, severe respiratory failure or associated with paradoxical vocal fold motion (PVFM) (91–93). Neonatal laryngospasm and unusual distribution of myotonia and other NDM signs have also been reported in several NDM patients, who have been shown to carry different *SCN4A* mutations, such as G1306E, I693T, A799S, N1297K, and T1313M (although not all patients that carry these mutations show childhood or neonatal respiratory problems) (78, 94–99). This expands the spectrum of phenotypes associated with mutations in the *SCN4A* gene. In addition to myotonic diseases associated with *SCN4A* mutations, other diseases, that are beyond the scope of this review, also present mutations in this gene, such as: hyperkalemic periodic paralysis without myotonia (HyperPP), hypokalemic periodic paralysis (HyppoPP), normokalemic periodic paralysis (NormoPP), and congenital myasthenic syndrome (CMS) (20, 82). Recently, it has also been suggested that a subset of cases with sudden infant death syndrome (SIDS) might be due to *SCN4A* mutations, as one report (and the only one thus far) has found novel or very rare functionally disruptive *SCN4A* genetic variants associated with SIDS, although the authors indicate that new studies in other populations are required to confirm their finding (100).

## Complex Genotype-Phenotype Relationships in Myotonic Channelopathies

In order to provide accurate prognostic information to the patients and families affected with a hereditary disease, it is essential to have appropriated genotype-phenotype relationships. In the case of the non-dystrophic myotonias, this has been extremely difficult mainly due to two factors: (1) the wide spectrum of symptoms and overlapping phenotypes present in both groups (Cl^−^ and Na^+^ myotonic channelopathies) (24, 25, 47), and (2) both genes, *CLCN1* and *SCN4A*, present high genotypic variability, with more than 200 or 65 different mutations already described, respectively (http://www.hgmd.cf.ac.uk/ac/index.php) (23, 43). Although many mutations cause a unique and reproducible phenotype in all patients, some *SCN4A* and *CLCN1* mutations cause similar phenotypes. Most worryingly, for several mutations, very different clinical phenotypes have been found in different carriers of the same mutation (23, 25, 47, 101), severely compromising the genotype-phenotype correlation in NDM. Another important limitation for the improvement of genotype-phenotype correlations has been the lack of a sufficient number of individuals carrying each mutation (23), in particular in the case of novel mutations [such as the very recent study that described seven novel *CLCN1* mutations (68)], which makes the situation even more complex, not without mentioning all those cases that show myotonia or a myotonia-like phenotype but in which the mutation has not been found. It is worth mentioning though, that correlation of mutations with the clinical phenotype gives insights into the pathophysiology of human channelopathies, and although some correlation exists between specific mutations and the associated clinical manifestations, this is vague (47).

The poor correlations are most evident in Cl^−^ channelopathies, where the same mutation can be inherited as dominant or recessive with different clinical manifestations, for example, F167L (39, 67, 68, 102, 103), A313T (67, 104), or W433R (44) (see Table 1). By recording and analyzing the ion currents of heterologously expressed mutant channels in different *in vitro* expression systems (*Xenopus* oocytes or HEK cells), much progress has been made in understanding how specific mutations affect the function of a particular ion channel, in providing insights onto the mechanism for the inheritance pattern, but also in the role that the voltage-gated ion channels play in excitable tissues (20, 25, 105). These analyses have contributed to improve to some extent, the clinical correlations through a better understanding of the channel dysfunction and its associated clinical picture. Nevertheless, of the more than 200 or 65 different Cl^−^ and Na^+^ mutations, only about 80 and 30 different mutations, respectively, have been functionally characterized (http://www.hgmd.cf.ac.uk/ac/index.php). However, these functional analyses have contributed to understand in a better way the recessive or dominant behavior of different *CLCN1* mutation than the overlapping phenotypes and clinical variability shown by some specific mutation (39, 67, 102, 103). For instance, in general, it has been reported that several recessively inherited *CLCN1* mutations show biophysical defects like reduced open probability, reduced single-channel conductance, or biochemical instability, in a manner that does impinge in a significant manner on the formation or function of heteromeric mutants/WT channels (13, 106, 107). A simple example are early stop codon mutations which do not result in the expression of ClC-1 subunits (108). In this regard, it has to be remembered that to provoke myotonia, G_Cl_ has to be lowered below roughly 30% (109–112). On the other hand, several dominantly inherited *CLCN1* mutations have been shown to exert *in vitro* a dominant negative effect in co-expression with WT (39, 58, 64, 107, 113, 114). In many cases the dominant negative effect is mediated by a shift of the open probability of the “common gate” to more positive voltages (107, 113, 114). These properties provide a rational to explain the dominant inheritance. However, this does not explain the clinical variability observed in both RMC and DMC. Interestingly, there is a group of 12 *CLCN1* mutations (see Table 2) in which the functional *in vitro* analyses have not been able to demonstrate differences with the WT channel, suggesting the presence of additional factors in skeletal muscle fibers, not present in oocytes/HEK cells, that are involved in the disease. Such factors could be related, for example, to subcellular targeting, a still open question for ClC-1 (18, 19). For these mutations, a powerful experimental approach would be the generation and analysis of knock-in mice carrying the myotonia-related *CLCN1* mutations, since myotonic mice are an established model for chloride channel myotonia (13, 115). Such knock-in mice could provide important information that cannot be obtained in heterologous expression systems. Additionally, patient derived induced pluripotent stem cells (iPSCs), combined with suitable myogenic differentiation (116), or the use of CRISPR/Cas approaches in order to correct (genome editing) specific mutations (followed by evaluating the off-target activities of CRISPR/Cas systems), might provide future avenues of studying the impact or correction of ion-channel mutations. However, in particular for *CLCN1*, these approaches are complicated by the fact that *CLCN1* expression (*in vivo*) requires fully differentiated and innervated muscle fibers (117). Importantly, many of these approaches also apply for the study of sodium channel mutations (see below).

**Table 1:** *CLCN1* mutations behaving as dominant/recessive.

**Mutation**	**Inheritance**	**Phenotype**	**Functional effects in heterologous expression systems (references of functional evidence)**	**References of genetic evidence**
c.501C>G, p.F167L	Recessive	Generalized Myotonia (compound heterozygous)	From very small shift of po to not different from WT (103, 118)	(39, 102)
	Dominant	Thomsen's like phenotype		(67, 103)
c.689G>A, p.G230E	Recessive	Generalized Myotonia (compound heterozygous)	Dominant negative effect, dramatic change in ion selectivity (58, 114)	(38)
	Dominant	Thomsen's disease		(104, 119)
c.803C>T, p.T268M	Recessive	Generalized Myotonia (compound heterozygous)	Changed po of the common gate (120)	(121)
	Dominant	Thomsen's disease		(119)
c.920T>C, p.F307S	Recessive	Generalized Myotonia (compound heterozygous)	Dominant negative effect, shifted the voltage dependence of po to positive potentials (74)	(122)
	Dominant	Thomsen's disease		(74)
c.937G>A, p.A313T	Recessive	Generalized Myotonia (compound heterozygous)	Drastically shifted the voltage dependence of po to positive potentials (74)	(104)
	Dominant	Thomsen's disease		
c.950G>A, p.R317Q	Recessive	Generalized Myotonia (compound heterozygous)	Shifted gating to positive potentials (113)	(62)
	Dominant	Thomsen's disease		(39)
c.1013G>A, p.R338Q	Recessive	Generalized Myotonia (compound heterozygous)	Shifted the voltage dependence of po to positive potentials (118)	(102)
	Dominant	Thomsen's disease		(38)
c.1297T>C, p.W433R	Recessive	Generalized Myotonia (compound heterozygous)	Not determined	(44)
	Dominant	Thomsen's disease		
c.1478C>A, p.A493E	Recessive	Generalized Myotonia (compound heterozygous and homozygous)	Unavailable	(123)
	Dominant	Thomsen's disease		(123)
c.1592C>T, p.A531V	Recessive	Generalized Myotonia (compound heterozygous)	Not determined	(5, 124)
	Dominant	Thomsen's disease		(5)
c.1667T>A, p.I556N	Recessive	Generalized Myotonia (homozygous)	Shifted the voltage dependence of po to positive potentials with minimal dominant negative effect (74)	(104)
	Dominant	Thomsen's disease (incomplete dominance)		
c.1936A>G, p.M646V	Recessive	Generalized Myotonia (compound heterozygous)	Unavailable	(123)
	Dominant	Thomsen's disease		(123)
c.2680C>T, p.R894X	Recessive	Generalized Myotonia (compound heterozygous)	Large reduction, but not complete abolition of chloride currents, and weak dominant effects (39)	(39, 121)
	Dominant	Thomsen's disease		(39, 102)

**Table 2:** *CLCN1* mutations behaving similar to ClC-1 WT in *in vitro* expression systems.

**Mutation**	**Phenotype**	**Functional analysis result**	**Heterologous expression system**	**References**
c.209C>T, p.S70L	Compatible with RMC	Macroscopic current amplitudes and current slopes comparable to WT	HEK293 cells	(125)
c.244A>G, p.T82A	Compatible with RMC	No effect on chloride currents, very similar to WT	tsA201 cells	(126)
c.313C>T, p.R105C	Compatible with RMC	Chloride currents similar to WT	HEK293 cells, *Xenopus* oocytes	(103, 106)
c.352T>G, p.W118G	Myotonia positive patients	Currents amplitudes similar to WT	HEK293 cells	(127)
c.449A>G, p.Y150C	Compatible with RMC	Currents indistinguishable from WT	*Xenopus* oocytes	(107)
c.501C>G, p.F167L	Compatible with RMC or DMC	Currents very similar to WT	HEK293 cells, *Xenopus* oocytes	(103, 106)
c.782A>G, p.Y261C	Compatible with RMC	Currents indistinguishable from WT	*Xenopus* oocytes	(107)
c.979G>A, p.V327I	Compatible with RMC	Currents very similar to WT	*Xenopus* oocytes	(61)
c.1357C>T, p.R453W	Compatible with RMC	No effect on chloride currents, very similar to WT	tsA201 cells	(126)
c.1412C>T, p.S471F	Myotonia positive patients	Electrophysiological parameters similar to WT	*Xenopus* oocytes	(128)
c.1883T>C, p.L628P	Compatible with RMC	Currents very similar to WT	tsA201 cells	(129)
c.2533G>A, p.G845S	Myotonia positive patients	Currents indistinguishable from WT ClC-1	tsA201 cells	(108)

*RMC, recessive myotonia congenita; DMC, dominant myotonia congenita; WT, wild type; NDM, non-dystrophic myotonia*.

Clinical correlations in sodium channel myotonic disorders are not as complex as the chloride channel myotonic disorders. As with MC, functional analyses in Na^+^ myotonic channelopathies have contributed to the understanding of the pathophysiological mechanism and improvement of clinical correlation, which are more accurate in this case. These analyses have been able to provide that Na^+^ channel mutations have four major effects on Nav1.4 function: (1) enhanced activation; (2) impaired slow inactivation; (3) impaired fast inactivation; and (4) accelerated recovery from fast inactivation. But, as with the *CLCN1* mutations, while these effects explain the dominant behavior of the mutations (dominant gain-of-function effect), they contribute little to the explanation of the clinical variability seen in this group of NDM. Nevertheless, it is well-accepted that: (1) a large fraction of persistent current and an incomplete slow inactivation of the channel may cause a strong long-lasting depolarization, providing the bases for weakness in HyperPP; (2) a slowing of fast inactivation and an incomplete closure of the channel may explain the paradoxical myotonia characteristic of PC; and (3) an increased persistent fraction and/or slowing of fast inactivation might explain the slight depolarization that causes myotonia in affected patients with SCM [reviewed in (47)]. But this does not explain the clinical variation observed in different patients, or even the overlapping symptoms seen with MC. In the case of *SCN4A* mutations, to the best of our knowledge, there have not been reported mutations that, functionally, behave as the WT channel.

Interestingly, there have been few patients carrying a *SCN4A* mutation who have been shown to carry a second mutation in *CLCN1* (see Table 3). These patients have shown an exacerbated or atypical Na^+^ channel disease phenotype, suggesting that both mutation may act synergistically to influence the clinical and neurophysiological phenotype observed in those patients (130). For many years, it was common practice to screen just one among *CLCN1* or *SCN4A* genes, based on the presumptive clinical phenotype. It is thus likely that a significant group of the first (or even some of recent cases) NMDs cases (with atypical/unclear phenotype, unclear inheritance pattern or dual inheritance-dominant or recessive) with molecular diagnosis, carry a second mutation in any of this ion channel genes, and, maybe even in other loci. Based on this and suggested by many authors, the recommendation is that in those NDM cases with atypical phenotypes or inheritance pattern, both *CLCN1* and *SCN4A* genes are screened. However, thanks to new technologies and price reduction, it might now be possible to carry out deep sequencing, such as whole genome sequencing or gene-targeted sequencing (by next generation sequencing-NGS), in these patients. These new approaches not only would allow to properly screen these genes and genotype NDM patients, but also, could contribute to identify gene modifiers that might be involved in modulating their phenotypes. In fact, very recently it was published the first report using whole genome sequencing for screening the *CLCN1* gene in RMC patients (131). These new approaches could be very useful to screen those patients with atypical phenotypes and those in which the effect of the identified mutation does not differ from the wild-type channel (see below).

**Table 3:** Simultaneous mutations in *CLCN1* and *SCN4A* in NDM patients showing an atypical phenotype.

**Mutations**	**Phenotype**	**Heterologous expression system/functional data**	**References**
*SCN4A* c.3917G>A, p.G1306E *CLCN1* c.1453A>G, p.M485V *SCN4A* c.4010G>C, p.R1337P *CLCN1* c.803C>T, p.T268M *SCN4A* c.2079T>G, p.I693M *CLCN1* c.2926C>T, p.Arg976X	Patient 1. Myotonic discharges with type II electrophysiology pattern. PC-like phenotype. Some signs of MC Patient 2. Myotonic discharges with type II electrophysiology pattern. PC-like phenotype. Some signs of MC Patient 3. Myotonic discharges with type III electrophysiology pattern. SCM-like phenotype. Some signs of MC	ND	(132)
*SCN4A* c.3870C>A, p.F1290L *CLCN1* c.2848 G>A, p.E950K	Myotonic discharges with type III electrophysiology pattern. SCM-like phenotype with periodic paralysis	HEK293 cells/*SCN4A =* enhanced activation-*CLCN1* = ND	(133)
*SCN4A* c.3890A>G, p.N1297S *CLCN1* c.501C>G, p.F167L	Mild NDM phenotype. SCM-like phenotype	HEK293 cells/*SCN4A* = impairment of fast and slow inactivation-*CLCN1* = ND	(134)
*SCN4A* c.665G**>**A, p.R222Q *CLCN1* c.1650G**>**A p.T550T	Patient with severe myotonia and without fulminant paralytic episodes	HEK293 cells/*SCN4A* = enhanced activation-*CLCN1* = ND	(135)

*MC, myotonia congenita; PC, paramyotonia congenita; SCM, sodium channel myotonias; NDM, non-dystrophic myotonia; ND, not determined*.

The molecular bases for the clinical variability present in myotonic channelopathies remain obscure. Yet, a deeper understanding is needed to improve genotype-phenotype correlations. Several hypotheses have been put forward to explain the clinical variability, which include: (a) differential allelic expression; (b) *trans*-acting genetic modifiers; (c) epigenetic, hormonal or environmental factors; and (d) dominance with low penetrance (20, 47, 136–138). These putatively acting (alone or in combination) mechanisms might contribute to explain why the same mutation causes different degrees of channel dysfunction in different patients (74), and therefore elicit a modulation in the NDM phenotype. Importantly, to the best of our knowledge, none of these possibilities has been established experimentally in NMD patients or are under current investigation.

The above considerations suggest that it is not only important to study as many individuals or mutations as possible, but also to try to obtain functional data for all mutations. In addition, deeper insight is expected from bioinformatics and structural approaches combined with information on nearby mutations, aided by the recently obtained 3D structure of the ClC-1 protein (139, 140). The results of these studies, in addition to explaining in a better way the different symptoms associated with particular mutations, would contribute to improving clinical correlations. Improvements in clinical tests, the recognition of the different phenotypes that result from particular mutations and the understanding of how a mutation affects the structure and function of the ion channel, together with genetic screening, is expected to improve clinical correlation in NDMs, which could ultimately be translated into better clinical management and to a better quality of life of affected patients and their families.

Finally, a solid knowledge of functional defects caused by specific mutations might help in the development of selective drugs targeted at a correction of such effects. For example, gating modifier drugs could be developed to invert the shift of the voltage-dependence of some dominant *CLCN1* mutations. Several small molecule compounds are indeed known to interfere with the open probability of ClC-1 channels, suggesting the principal feasibility of such an approach (141, 142). In particular, derivatives of clofibric acid have been shown to shift the voltage-dependence of ClC-1 and the related ClC-0 to more positive voltages (141–143). The development of novel molecules that specifically bind to and stabilize the open state of the channel could be aided by the recent determination of the structure of ClC-1 (139, 140). Another possible line of intervention regards patients with mutations that lead to protein folding defects. Such defects might be treated with small molecules that act as “correctors.” Such an approach is already in clinical use in other diseases, such as cystic fibrosis (144). Since correctors are assumed to stabilize the folded state of the channel, it might be tempting to start the search of correctors using ClC-1 inhibitors, like 9-anthrazene carboxylic acid (9AC) (143). However, all known ClC-1 inhibitors are of rather low affinity and quite unspecific (8). Therefore, such correctors are likely only to be discovered using high-throughput screening, as done for the most common CFTR mutation causing cystic fibrosis (118). Indeed, generic correctors, such as 4-phenylbutyrate, miglustat, and sildenafil have proven unsuccessful for cystic fibrosis (118).

For preparing this review, we (both authors) have used our personal historical literature database of publications regarding *CLCN1* and *SCN4A* related myotonia, as well as PubMed searches using the keywords “myotonia AND (CLCN1/CLC-1, SCN4A/Nav1.4, apnea, laryngospasm).”

## Author Contributions

All authors listed have made a substantial, direct and intellectual contribution to the work, and approved it for publication.

### Conflict of Interest

The authors declare that the research was conducted in the absence of any commercial or financial relationships that could be construed as a potential conflict of interest. The reviewer DF and handling editor declared their shared affiliation at the time of the review.

## References

[1] Emery AE. The muscular dystrophies. Lancet. (2002) 359:687–95. 10.1016/S0140-6736(02)07815-7 11879882

[2] Mercuri E Muntoni F. Muscular dystrophies. Lancet. (2013) 381:845–60. 10.1016/S0140-6736(12)61897-2 23465426

[3] Rahimov F Kunkel LM. The cell biology of disease: cellular and molecular mechanisms underlying muscular dystrophy. J Cell Biol. (2013) 201:499–510. 10.1083/jcb.201212142 23671309 PMC3653356

[4] Colding-Jorgensen E. Phenotypic variability in myotonia congenita. Muscle Nerve. (2005) 32:19–34. 10.1002/mus.20295 15786415

[5] Sun C Tranebjaerg L Torbergsen T Holmgren G Van Ghelue M. Spectrum of CLCN1 mutations in patients with myotonia congenita in Northern Scandinavia. Eur J Hum Genet. (2001) 9:903–9. 10.1038/sj.ejhg.5200736 11840191

[6] Pedersen TH Riisager A de Paoli FV Chen TY Nielsen OB. Role of physiological ClC-1 Cl- ion channel regulation for the excitability and function of working skeletal muscle. J Gen Physiol. (2016) 147:291–308. 10.1085/jgp.201611582 27022190 PMC4810071

[7] Stauber T Weinert S Jentsch TJ. Cell biology and physiology of CLC chloride channels and transporters. Compr Physiol. (2012) 2:1701–44. 10.1002/cphy.c110038 23723021

[8] Jentsch TJ Pusch M. CLC chloride channels and transporters: structure, function, physiology, and disease. Physiol Rev. (2018) 98:1493–590. 10.1152/physrev.00047.2017 29845874

[9] Jentsch TJ Steinmeyer K Schwarz G. Primary structure of Torpedo marmorata chloride channel isolated by expression cloning in *Xenopus* oocytes. Nature. (1990) 348:510–4. 10.1038/348510a0 2174129

[10] Koch MC Steinmeyer K Lorenz C Ricker K Wolf F Otto M. The skeletal muscle chloride channel in dominant and recessive human myotonia. Science. (1992) 257:797–800. 10.1126/science.1379744 1379744

[11] Miller C. ClC chloride channels viewed through a transporter lens. Nature. (2006) 440:484–9. 10.1038/nature04713 16554809

[12] Bryant SH Morales-Aguilera A. Chloride conductance in normal and myotonic muscle fibres and the action of monocarboxylic aromatic acids. J Physiol. (1971) 219:367–83. 10.1113/jphysiol.1971.sp009667 5316641 PMC1331636

[13] Steinmeyer K Klocke R Ortland C Gronemeier M Jockusch H Grunder S. Inactivation of muscle chloride channel by transposon insertion in myotonic mice. Nature. (1991) 354:304–8. 10.1038/354304a0 1659665

[14] Steinmeyer K Ortland C Jentsch TJ. Primary structure and functional expression of a developmentally regulated skeletal muscle chloride channel. Nature. (1991) 354:301–4. 10.1038/354301a0 1659664

[15] Bretag AH. Muscle chloride channels. Physiol Rev. (1987) 67:618–724. 10.1152/physrev.1987.67.2.618 2436244

[16] Lamb GD Murphy RM Stephenson DG. On the localization of ClC-1 in skeletal muscle fibers. J Gen Physiol. (2011) 137:327–9. 10.1085/jgp.201010580 21357735 PMC3047610

[17] Zifarelli G Pusch M. Relaxing messages from the sarcolemma. J Gen Physiol. (2010) 136:593–6. 10.1085/jgp.201010567 21078870 PMC2995151

[18] DiFranco M Herrera A Vergara JL. Chloride currents from the transverse tubular system in adult mammalian skeletal muscle fibers. J Gen Physiol. (2011) 137:21–41. 10.1085/jgp.201010496 21149546 PMC3010054

[19] Lueck JD Rossi AE Thornton CA Campbell KP Dirksen RT. Sarcolemmal-restricted localization of functional ClC-1 channels in mouse skeletal muscle. J Gen Physiol. (2010) 136:597–613. 10.1085/jgp.201010526 21078869 PMC2995150

[20] Cannon SC. Channelopathies of skeletal muscle excitability. Compr Physiol. (2015) 5:761–90. 10.1002/cphy.c140062 25880512 PMC4754081

[21] Kent-Braun JA Fitts RH Christie A. Skeletal muscle fatigue. Compr Physiol. (2012) 2:997–1044. 10.1002/cphy.c110029 23798294

[22] Trivedi JR Bundy B Statland J Salajegheh M Rayan DR Venance SL. Non-dystrophic myotonia: prospective study of objective and patient reported outcomes. Brain. (2013) 136(Pt 7):2189–200. 10.1093/brain/awt133 23771340 PMC3692030

[23] Matthews E Fialho D Tan SV Venance SL Cannon SC Sternberg D. The non-dystrophic myotonias: molecular pathogenesis, diagnosis and treatment. Brain. (2010) 133(Pt 1):9–22. 10.1093/brain/awp294 19917643 PMC2801326

[24] Heatwole CR Moxley RT III. The nondystrophic myotonias. Neurotherapeutics. (2007) 4:238–51. 10.1016/j.nurt.2007.01.012 17395134

[25] Platt D Griggs R. Skeletal muscle channelopathies: new insights into the periodic paralyses and nondystrophic myotonias. Curr Opin Neurol. (2009) 22:524–31. 10.1097/WCO.0b013e32832efa8f 19571750 PMC2763141

[26] AANEM American Association of Electrodiagnostic Medicine glossary of terms in electrodiagnostic medicine. Muscle Nerve Suppl. (2001) 10:S1–50. 11603366

[27] Hudson AJ Ebers GC Bulman DE. The skeletal muscle sodium and chloride channel diseases. Brain. (1995) 118(Pt 2):547–63. 10.1093/brain/118.2.547 7735894

[28] Ricker K Hertel G Langscheid K Stodieck G Myotonia not aggravated by cooling. Force and relaxation of the adductor pollicis in normal subjects and in myotonia as compared to paramyotonia. J Neurol. (1977) 216:9–20. 10.1007/BF00312810 72799

[29] Streib EW AAEE minimonograph #27: differential diagnosis of myotonic syndromes. Muscle Nerve. (1987) 10:603–15. 10.1002/mus.880100704 3309651

[30] Morales F Cuenca P Aspectos geneticos y moleculares de las enfermedades miotonicas. Rev Neurol. (2004) 38:668–74. 10.33588/rn.3807.2003483 15098190

[31] Jurkat-Rott K Lerche H Lehmann-Horn F. Skeletal muscle channelopathies. J Neurol. (2002) 249:1493–502. 10.1007/s00415-002-0871-5 12420087

[32] Lehmann-Horn F Jurkat-Rott K Rudel R. Periodic paralysis: understanding channelopathies. Curr Neurol Neurosci Rep. (2002) 2:61–9. 10.1007/s11910-002-0055-9 11898585

[33] Goodman BE. Channels active in the excitability of nerves and skeletal muscles across the neuromuscular junction: basic function and pathophysiology. Adv Physiol Educ. (2008) 32:127–35. 10.1152/advan.00091.2007 18539851

[34] Imbrici P Altamura C Pessia M Mantegazza R Desaphy JF Camerino DC. ClC-1 chloride channels: state-of-the-art research and future challenges. Front Cell Neurosci. (2015) 9:156. 10.3389/fncel.2015.00156 25964741 PMC4410605

[35] Thomsen J Tonische Krampfe in willkurlich beweglichen Muskeln in Folge von ererbter psychischer Disposition (Ataxia muscularis?). Arch Psychiatr Nervenkr. (1876) 6:702–18.

[36] Westphal C Demonstration zweier Falle von Thomsen'scher Krankheit. Berl Klin Wschr. (1883) 20:153.

[37] Becker PE Zur Genetik der Myotonien. In: Kuhn E editor. Progressive Muskeldystrophie–Myotonie–Myasthenie. Berlin: Springer Verlag (1966). p. 247–55.

[38] Zhang J George AL Jr Griggs RC Fouad GT Roberts J Kwiecinski H. Mutations in the human skeletal muscle chloride channel gene (CLCN1) associated with dominant and recessive myotonia congenita. Neurology. (1996) 47:993–8. 10.1212/WNL.47.4.993 8857733

[39] Meyer-Kleine C Steinmeyer K Ricker K Jentsch TJ Koch MC. Spectrum of mutations in the major human skeletal muscle chloride channel gene (CLCN1) leading to myotonia. Am J Hum Genet. (1995) 57:1325–34. 8533761 PMC1801423

[40] Lehmann-Horn F Jurkat-Rott K. Voltage-gated ion channels and hereditary disease. Physiol Rev. (1999) 79:1317–72. 10.1152/physrev.1999.79.4.1317 10508236

[41] Pusch M. Myotonia caused by mutations in the muscle chloride channel gene CLCN1. Hum Mutat. (2002) 19:423–34. 10.1002/humu.10063 11933197

[42] Horga A Raja Rayan DL Matthews E Sud R Fialho D Durran SC. Prevalence study of genetically defined skeletal muscle channelopathies in England. Neurology. (2013) 80:1472–5. 10.1212/WNL.0b013e31828cf8d0 23516313 PMC3662361

[43] Lossin C George AL Jr. Myotonia congenita. Adv Genet. (2008) 63:25–55. 10.1016/S0065-2660(08)01002-X 19185184

[44] Dupre N Chrestian N Bouchard JP Rossignol E Brunet D Sternberg D. Clinical, electrophysiologic, and genetic study of non-dystrophic myotonia in French-Canadians. Neuromuscul Disord. (2009) 19:330–4. 10.1016/j.nmd.2008.01.007 18337100

[45] Koch MC Ricker K Otto M Wolf F Zoll B Lorenz C. Evidence for genetic homogeneity in autosomal recessive generalised myotonia (Becker). J Med Genet. (1993) 30:914–7. 10.1136/jmg.30.11.914 8301644 PMC1016598

[46] Mankodi A. Myotonic disorders. Neurol India. (2008) 56:298–304. 10.4103/0028-3886.43448 18974556

[47] Vicart S Sternberg D Fontaine B Meola G. Human skeletal muscle sodium channelopathies. Neurol Sci. (2005) 26:194–202. 10.1007/s10072-005-0461-x 16193245

[48] Fournier E Arzel M Sternberg D Vicart S Laforet P Eymard B. Electromyography guides toward subgroups of mutations in muscle channelopathies. Ann Neurol. (2004) 56:650–61. 10.1002/ana.20241 15389891

[49] Ptacek LJ Tawil R Griggs RC Meola G McManis P Barohn RJ. Sodium channel mutations in acetazolamide-responsive myotonia congenita, paramyotonia congenita, and hyperkalemic periodic paralysis. Neurology. (1994) 44:1500–3. 10.1212/WNL.44.8.1500 8058156

[50] Sansone VA. The Dystrophic and Nondystrophic Myotonias. Continuum (Minneap Minn). (2016) 22:1889–915. 10.1212/CON.0000000000000414 27922499

[51] Von Eulenburg A Über eine familiäre durch 6 Generationen verfolgbare Form kongenitaler Paramyotonie. Neurol Zentralbl. (1986) 5:265–72.

[52] Jurkat-Rott K Lehmann-Horn F. Genotype-phenotype correlation and therapeutic rationale in hyperkalemic periodic paralysis. Neurotherapeutics. (2007) 4:216–24. 10.1016/j.nurt.2007.02.001 17395131

[53] Weber F Jurkat-Rott K Lehmann-Horn F. Hyperkalemic periodic paralysis. In: Adam MP Ardinger HH Pagon RA Wallace SE Bean LJH Stephens K ., editors. GeneReviews^®^. Seattle, WA (1993). Available online at: https://www.ncbi.nlm.nih.gov/books/NBK1496

[54] Charles G Zheng C Lehmann-Horn F Jurkat-Rott K Levitt J. Characterization of hyperkalemic periodic paralysis: a survey of genetically diagnosed individuals. J Neurol. (2013) 260:2606–13. 10.1007/s00415-013-7025-9 23884711 PMC3824221

[55] Cannon SC. Sodium Channelopathies of Skeletal Muscle. Handb Exp Pharmacol. (2018) 246:309–30. 10.1007/164_2017_52 28939973 PMC5866235

[56] Becker PE Myotonia Congenita and Syndromes Associated with Myotonia. Sttutgart: Thieme (1977).

[57] George AL Jr Crackower MA Abdalla JA Hudson AJ Ebers GC. Molecular basis of Thomsen's disease (autosomal dominant myotonia congenita). Nat Genet. (1993) 3:305–10. 10.1038/ng0493-305 7981750

[58] Steinmeyer K Lorenz C Pusch M Koch MC Jentsch TJ. Multimeric structure of ClC-1 chloride channel revealed by mutations in dominant myotonia congenita (Thomsen). Embo J. (1994) 13:737–43. 10.1002/j.1460-2075.1994.tb06315.x 8112288 PMC394869

[59] Abdalla JA Casley WL Cousin HK Hudson AJ Murphy EG Cornelis FC. Linkage of Thomsen disease to the T-cell-receptor beta (TCRB) locus on chromosome 7q35. Am J Hum Genet. (1992) 51:579–84. 1386711 PMC1682708

[60] Grunnet M Jespersen T Colding-Jorgensen E Schwartz M Klaerke DA Vissing J. Characterization of two new dominant ClC-1 channel mutations associated with myotonia. Muscle Nerve. (2003) 28:722–32. 10.1002/mus.10501 14639587

[61] Lorenz C Meyer-Kleine C Steinmeyer K Koch MC Jentsch TJ. Genomic organization of the human muscle chloride channel CIC-1 and analysis of novel mutations leading to Becker-type myotonia. Hum Mol Genet. (1994) 3:941–6. 10.1093/hmg/3.6.941 7951242

[62] Esteban J Neumeyer AM McKenna-Yasek D Brown RH. Identification of two mutations and a polymorphism in the chloride channel CLCN-1 in patients with Becker's generalized myotonia. Neurogenetics. (1998) 1:185–8. 10.1007/s100480050027 10737121

[63] Zhang J Sanguinetti MC Kwiecinski H Ptacek LJ. Mechanism of inverted activation of ClC-1 channels caused by a novel myotonia congenita mutation. J Biol Chem. (2000) 275:2999–3005. 10.1074/jbc.275.4.2999 10644771

[64] Saviane C Conti F Pusch M. The muscle chloride channel ClC-1 has a double-barreled appearance that is differentially affected in dominant and recessive myotonia. J Gen Physiol. (1999) 113:457–68. 10.1085/jgp.113.3.457 10051520 PMC2222904

[65] Middleton RE Pheasant DJ Miller C. Homodimeric architecture of a ClC-type chloride ion channel. Nature. (1996) 383:337–40. 10.1038/383337a0 8848046

[66] Ludewig U Pusch M Jentsch TJ. Two physically distinct pores in the dimeric ClC-0 chloride channel. Nature. (1996) 383:340–3. 10.1038/383340a0 8848047

[67] Fialho D Schorge S Pucovska U Davies NP Labrum R Haworth A. Chloride channel myotonia: exon 8 hot-spot for dominant-negative interactions. Brain. (2007) 130(Pt 12):3265–74. 10.1093/brain/awm248 17932099

[68] Milla CP De Castro CP Gomez-Gonzalez C Martinez-Montero P Pascual Pascual SI Molano Mateos J. Myotonia congenita: mutation spectrum of CLCN1 in Spanish patients. J Genet. (2019) 98:71. 10.1007/s12041-019-1115-0 31544778

[69] Raja Rayan DL Haworth A Sud R Matthews E Fialho D Burge J. A new explanation for recessive myotonia congenita: exon deletions and duplications in CLCN1. Neurology. (2012) 78:1953–8. 10.1212/WNL.0b013e318259e19c 22649220 PMC3369509

[70] Tang CY Chen TY. Physiology and pathophysiology of CLC-1: mechanisms of a chloride channel disease, myotonia. J Biomed Biotechnol. (2011) 2011:685328. 10.1155/2011/685328 22187529 PMC3237021

[71] de Diego C Gamez J Plassart-Schiess E Lasa A Del Rio E Cervera C. Novel mutations in the muscle chloride channel CLCN1 gene causing myotonia congenita in Spanish families. J Neurol. (1999) 246:825–9. 10.1007/s004150050462 10525982

[72] Koty PP Pegoraro E Hobson G Marks HG Turel A Flagler D. Myotonia and the muscle chloride channel: dominant mutations show variable penetrance and founder effect. Neurology. (1996) 47:963–8. 10.1212/WNL.47.4.963 8857727

[73] Brugnoni R Kapetis D Imbrici P Pessia M Canioni E Colleoni L. A large cohort of myotonia congenita probands: novel mutations and a high-frequency mutation region in exons 4 and 5 of the CLCN1 gene. J Hum Genet. (2013) 58:581–7. 10.1038/jhg.2013.58 23739125

[74] Kubisch C Schmidt-Rose T Fontaine B Bretag AH Jentsch TJ. ClC-1 chloride channel mutations in myotonia congenita: variable penetrance of mutations shifting the voltage dependence. Hum Mol Genet. (1998) 7:1753–60. 10.1093/hmg/7.11.1753 9736777

[75] Koch MC Ricker K Otto M Grimm T Hoffman EP Rudel R. Confirmation of linkage of hyperkalaemic periodic paralysis to chromosome 17. J Med Genet. (1991) 28:583–6. 10.1136/jmg.28.9.583 1683408 PMC1015786

[76] Ptacek LJ George AL Jr Barchi RL Griggs RC Riggs JE Robertson M. Identification of a mutation in the gene causing hyperkalemic periodic paralysis. Cell. (1991) 67:1021–7. 10.1016/0092-8674(91)90374-8 1659948

[77] Ptacek LJ Trimmer JS Agnew WS Roberts JW Petajan JH Leppert M. Paramyotonia congenita and hyperkalemic periodic paralysis map to the same sodium-channel gene locus. Am J Hum Genet. (1991) 49:851–4. 1654742 PMC1683172

[78] Lerche H Heine R Pika U George AL Jr Mitrovic N Browatzki M. Human sodium channel myotonia: slowed channel inactivation due to substitutions for a glycine within the III-IV linker. J Physiol. (1993) 470:13–22. 10.1113/jphysiol.1993.sp019843 8308722 PMC1143902

[79] Chahine M George AL Jr Zhou M Ji S Sun W Barchi RL. Sodium channel mutations in paramyotonia congenita uncouple inactivation from activation. Neuron. (1994) 12:281–94. 10.1016/0896-6273(94)90271-2 8110459

[80] Lehmann-Horn F Rudel R Ricker K. Membrane defects in paramyotonia congenita (Eulenburg). Muscle Nerve. (1987) 10:633–41. 10.1002/mus.880100709 3657849

[81] Hanna MG. Genetic neurological channelopathies. Nat Clin Pract Neurol. (2006) 2:252–63. 10.1038/ncpneuro0178 16932562

[82] Jurkat-Rott K Holzherr B Fauler M Lehmann-Horn F Sodium channelopathies of skeletal muscle result from gain or loss of function. Pflugers Arch. (2010) 460:239–48. 10.1007/s00424-010-0814-4 20237798 PMC2883924

[83] Jurkat-Rott K Lehmann-Horn F. Muscle channelopathies and critical points in functional and genetic studies. J Clin Invest. (2005) 115:2000–9. 10.1172/JCI25525 16075040 PMC1180551

[84] Cannon SC. Pathomechanisms in channelopathies of skeletal muscle and brain. Annu Rev Neurosci. (2006) 29:387–415. 10.1146/annurev.neuro.29.051605.112815 16776591

[85] Fialho D Hanna MG. Periodic paralysis. Handb Clin Neurol. (2007) 86:77–106. 10.1016/S0072-9752(07)86004-0 18808996

[86] Huang W Liu M Yan SF Yan N. Structure-based assessment of disease-related mutations in human voltage-gated sodium channels. Protein Cell. (2017) 8:401–38. 10.1007/s13238-017-0372-z 28150151 PMC5445024

[87] Kubota T Roca X Kimura T Kokunai Y Nishino I Sakoda S. A mutation in a rare type of intron in a sodium-channel gene results in aberrant splicing and causes myotonia. Hum Mutat. (2011) 32:773–82. 10.1002/humu.21501 21412952 PMC4109284

[88] Stenson PD Ball EV Mort M Phillips AD Shiel JA Thomas NS. Human gene mutation database (HGMD): 2003 update. Hum Mutat. (2003) 21:577–81. 10.1002/humu.10212 12754702

[89] Lehmann-Horn F Kuther G Ricker K Grafe P Ballanyi K Rudel R. Adynamia episodica hereditaria with myotonia: a non-inactivating sodium current and the effect of extracellular pH. Muscle Nerve. (1987) 10:363–74. 10.1002/mus.880100414 3587272

[90] Davies NP Eunson LH Gregory RP Mills KR Morrison PJ Hanna MG. Clinical, electrophysiological, and molecular genetic studies in a new family with paramyotonia congenita. J Neurol Neurosurg Psychiatry. (2000) 68:504–7. 10.1136/jnnp.68.4.504 10727489 PMC1736851

[91] Turkdogan D Matthews E Usluer S Gundogdu A Uluc K Mannikko R. Possible role of SCN4A skeletal muscle mutation in apnea during seizure. Epilepsia Open. (2019) 4:498–503. 10.1002/epi4.12347 31440732 PMC6698682

[92] Pechmann A Eckenweiler M Schorling D Stavropoulou D Lochmuller H Kirschner J. *De novo* variant in SCN4A causes neonatal sodium channel myotonia with general muscle stiffness and respiratory failure. Neuromuscul Disord. (2019) 29:907–9. 10.1016/j.nmd.2019.09.001 31732390

[93] Purkey MR Valika T. A unique presentation and etiology of neonatal paradoxical vocal fold motion. Int J Pediatr Otorhinolaryngol. (2019) 125:199–200. 10.1016/j.ijporl.2019.07.011 31382107

[94] Lehmann-Horn F D'Amico A Bertini E Lomonaco M Merlini L Nelson KR. Myotonia permanens with Nav1.4-G1306E displays varied phenotypes during course of life. Acta Myol. (2017) 36:125–34. 29774303 PMC5953224

[95] Caietta E Milh M Sternberg D Lepine A Boulay C McGonigal A. Diagnosis and outcome of SCN4A-related severe neonatal episodic laryngospasm (SNEL): 2 new cases. Pediatrics. (2013) 132:e784–7. 10.1542/peds.2012-3065 23958773

[96] Singh RR Tan SV Hanna MG Robb SA Clarke A Jungbluth H. Mutations in SCN4A: a rare but treatable cause of recurrent life-threatening laryngospasm. Pediatrics. (2014) 134:e1447–50. 10.1542/peds.2013-3727 25311598

[97] Gay S Dupuis D Faivre L Masurel-Paulet A Labenne M Colombani M. Severe neonatal non-dystrophic myotonia secondary to a novel mutation of the voltage-gated sodium channel (SCN4A) gene. Am J Med Genet A. (2008) 146A:380–3. 10.1002/ajmg.a.32141 18203179

[98] Matthews E Guet A Mayer M Vicart S Pemble S Sternberg D. Neonatal hypotonia can be a sodium channelopathy: recognition of a new phenotype. Neurology. (2008) 71:1740–2. 10.1212/01.wnl.0000335269.21550.0e 19015492 PMC2676969

[99] Lion-Francois L Mignot C Vicart S Manel V Sternberg D Landrieu P. Severe neonatal episodic laryngospasm due to *de novo* SCN4A mutations: a new treatable disorder. Neurology. (2010) 75:641–5. 10.1212/WNL.0b013e3181ed9e96 20713951

[100] Mannikko R Wong L Tester DJ Thor MG Sud R Kullmann DM. Dysfunction of NaV1.4, a skeletal muscle voltage-gated sodium channel, in sudden infant death syndrome: a case-control study. Lancet. (2018) 391:1483–92. 10.1016/S0140-6736(18)30021-7 29605429 PMC5899997

[101] Lee SC Kim HS Park YE Choi YC Park KH Kim DS. Clinical diversity of SCN4A-mutation-associated skeletal muscle sodium channelopathy. J Clin Neurol. (2009) 5:186–91. 10.3988/jcn.2009.5.4.186 20076800 PMC2806541

[102] George AL Jr Sloan-Brown K Fenichel GM Mitchell GA Spiegel R Pascuzzi RM. Nonsense and missense mutations of the muscle chloride channel gene in patients with myotonia congenita. Hum Mol Genet. (1994) 3:2071–2. 7874130

[103] Vindas-Smith R Fiore M Vasquez M Cuenca P Del Valle G Lagostena L. Identification and functional characterization of CLCN1 mutations found in nondystrophic myotonia patients. Hum Mutat. (2016) 37:74–83. 10.1002/humu.22916 26510092

[104] Plassart-Schiess E Gervais A Eymard B Lagueny A Pouget J Warter JM. Novel muscle chloride channel (CLCN1) mutations in myotonia congenita with various modes of inheritance including incomplete dominance and penetrance. Neurology. (1998) 50:1176–9. 10.1212/WNL.50.4.1176 9566422

[105] Planells-Cases R Jentsch TJ. Chloride channelopathies. Biochim Biophys Acta. (2009) 1792:173–89. 10.1016/j.bbadis.2009.02.002 19708126

[106] Desaphy JF Gramegna G Altamura C Dinardo MM Imbrici P George AL Jr. Functional characterization of ClC-1 mutations from patients affected by recessive myotonia congenita presenting with different clinical phenotypes. Exp Neurol. (2013) 248:530–40. 10.1016/j.expneurol.2013.07.018 23933576 PMC3781327

[107] Wollnik B Kubisch C Steinmeyer K Pusch M. Identification of functionally important regions of the muscular chloride channel CIC-1 by analysis of recessive and dominant myotonic mutations. Hum Mol Genet. (1997) 6:805–11. 10.1093/hmg/6.5.805 9158157

[108] Ulzi G Lecchi M Sansone V Redaelli E Corti E Saccomanno D. Myotonia congenita: novel mutations in CLCN1 gene and functional characterizations in Italian patients. J Neurol Sci. (2012) 318:65–71. 10.1016/j.jns.2012.03.024 22521272

[109] Barchi RL. Myotonia. An evaluation of the chloride hypothesis. Arch Neurol. (1975) 32:175–80. 10.1001/archneur.1975.00490450055007 1119960

[110] Kwiecinski H Lehmann-Horn F Rudel R. Drug-induced myotonia in human intercostal muscle. Muscle Nerve. (1988) 11:576–81. 10.1002/mus.880110609 2455224

[111] Adrian RH Marshall MW. Action potentials reconstructed in normal and myotonic muscle fibres. J Physiol. (1976) 258:125–43. 10.1113/jphysiol.1976.sp011410 940049 PMC1308963

[112] Furman RE Barchi RL. The pathophysiology of myotonia produced by aromatic carboxylic acids. Ann Neurol. (1978) 4:357–65. 10.1002/ana.410040411 727740

[113] Pusch M Steinmeyer K Koch MC Jentsch TJ. Mutations in dominant human myotonia congenita drastically alter the voltage dependence of the CIC-1 chloride channel. Neuron. (1995) 15:1455–63. 10.1016/0896-6273(95)90023-3 8845168

[114] Fahlke C Beck CL George AL Jr. A mutation in autosomal dominant myotonia congenita affects pore properties of the muscle chloride channel. Proc Natl Acad Sci USA. (1997) 94:2729–34. 10.1073/pnas.94.6.2729 9122265 PMC20158

[115] Reininghaus J Fuchtbauer EM Bertram K Jockusch H. The myotonic mouse mutant ADR: physiological and histochemical properties of muscle. Muscle Nerve. (1988) 11:433–9. 10.1002/mus.880110504 2967431

[116] Jiwlawat N Lynch E Jeffrey J Van Dyke JM Suzuki M. Current progress and challenges for skeletal muscle differentiation from human pluripotent stem cells using transgene-free approaches. Stem Cells Int. (2018) 2018:6241681. 10.1155/2018/6241681 29760730 PMC5924987

[117] Klocke R Steinmeyer K Jentsch TJ Jockusch H. Role of innervation, excitability, and myogenic factors in the expression of the muscular chloride channel ClC-1. A study on normal and myotonic muscle. J Biol Chem. (1994) 269:27635–9. 7961681

[118] Zhang J Bendahhou S Sanguinetti MC Ptacek LJ. Functional consequences of chloride channel gene (CLCN1) mutations causing myotonia congenita. Neurology. (2000) 54:937–42. 10.1212/WNL.54.4.937 10690989

[119] Brugnoni R Galantini S Confalonieri P Balestrini MR Cornelio F Mantegazza R. Identification of three novel mutations in the major human skeletal muscle chloride channel gene (CLCN1), causing myotonia congenita. Hum Mutat. (1999) 14:447. 10.1002/(SICI)1098-1004(199911)14:5<447::AID-HUMU13>3.0.CO;2-Z 10533075

[120] Duffield M Rychkov G Bretag A Roberts M. Involvement of helices at the dimer interface in ClC-1 common gating. J Gen Physiol. (2003) 121:149–61. 10.1085/jgp.20028741 12566541 PMC2217322

[121] Deymeer F Cakirkaya S Serdaroglu P Schleithoff L Lehmann-Horn F Rudel R. Transient weakness and compound muscle action potential decrement in myotonia congenita. Muscle Nerve. (1998) 21:1334–7. 10.1002/(SICI)1097-4598(199810)21:10<1334::AID-MUS16>3.0.CO;2-1 9736066

[122] Colding-Jorgensen E Dun OM Schwartz M Vissing J. Decrement of compound muscle action potential is related to mutation type in myotonia congenita. Muscle Nerve. (2003) 27:449–55. 10.1002/mus.10347 12661046

[123] Ivanova EA Dadali EL Fedotov VP Kurbatov SA Rudenskaia GE Proskokova TN. The spectrum of CLCN1 gene mutations in patients with nondystrophic Thomsen's and Becker's myotonias. Genetika. (2012) 48:1113–23. 10.1134/S1022795412090049 23113340

[124] Papponen H Toppinen T Baumann P Myllyla V Leisti J Kuivaniemi H. Founder mutations and the high prevalence of myotonia congenita in northern Finland. Neurology. (1999) 53:297–302. 10.1212/WNL.53.2.297 10430417

[125] Ronstedt K Sternberg D Detro-Dassen S Gramkow T Begemann B Becher T. Impaired surface membrane insertion of homo- and heterodimeric human muscle chloride channels carrying amino-terminal myotonia-causing mutations. Sci Rep. (2015) 5:15382. 10.1038/srep15382 26502825 PMC4621517

[126] Portaro S Altamura C Licata N Camerino GM Imbrici P Musumeci O. Clinical, molecular, and functional characterization of CLCN1 mutations in three families with recessive myotonia congenita. Neuromolecular Med. (2015) 17:285–96. 10.1007/s12017-015-8356-8 26007199 PMC4534513

[127] Raheem O Penttila S Suominen T Kaakinen M Burge J Haworth A. New immunohistochemical method for improved myotonia and chloride channel mutation diagnostics. Neurology. (2012) 79:2194–200. 10.1212/WNL.0b013e31827595e2 23152584 PMC3570820

[128] Lin MJ You TH Pan H Hsiao KM. Functional characterization of CLCN1 mutations in Taiwanese patients with myotonia congenita via heterologous expression. Biochem Biophys Res Commun. (2006) 351:1043–7. 10.1016/j.bbrc.2006.10.158 17097617

[129] Imbrici P Maggi L Mangiatordi GF Dinardo MM Altamura C Brugnoni R. ClC-1 mutations in myotonia congenita patients: insights into molecular gating mechanisms and genotype-phenotype correlation. J Physiol. (2015) 593:4181–99. 10.1113/JP270358 26096614 PMC4594292

[130] Skov M Riisager A Fraser JA Nielsen OB Pedersen TH. Extracellular magnesium and calcium reduce myotonia in ClC-1 inhibited rat muscle. Neuromuscul Disord. (2013) 23:489–502. 10.1016/j.nmd.2013.03.009 23623567

[131] Wei Z Huaxing M Xiaomei W Juan W Xueli C Jing Z. Identification of two novel compound heterozygous CLCN1 mutations associated with autosomal recessive myotonia congenita. Neurol Res. (2019) 41:1069–74. 10.1080/01616412.2019.1672392 31566103

[132] Furby A Vicart S Camdessanche JP Fournier E Chabrier S Lagrue E. Heterozygous CLCN1 mutations can modulate phenotype in sodium channel myotonia. Neuromuscul Disord. (2014) 24:953–9. 10.1016/j.nmd.2014.06.439 25088311

[133] Kato H Kokunai Y Dalle C Kubota T Madokoro Y Yuasa H. A case of non-dystrophic myotonia with concomitant mutations in the SCN4A and CLCN1 genes. J Neurol Sci. (2016) 369:254–8. 10.1016/j.jns.2016.08.030 27653901

[134] Maggi L Ravaglia S Farinato A Brugnoni R Altamura C Imbrici P. Coexistence of CLCN1 and SCN4A mutations in one family suffering from myotonia. Neurogenetics. (2017) 18:219–25. 10.1007/s10048-017-0525-5 28993909

[135] Thor MG Vivekanandam V Sampedro-Castaneda M Tan SV Suetterlin K Sud R. Myotonia in a patient with a mutation in an S4 arginine residue associated with hypokalaemic periodic paralysis and a concomitant synonymous CLCN1 mutation. Sci Rep. (2019) 9:17560. 10.1038/s41598-019-54041-0 31772215 PMC6879752

[136] Duno M Colding-Jorgensen E Grunnet M Jespersen T Vissing J Schwartz M. Difference in allelic expression of the CLCN1 gene and the possible influence on the myotonia congenita phenotype. Eur J Hum Genet. (2004) 12:738–43. 10.1038/sj.ejhg.5201218 15162127

[137] Modoni A D'Amico A Dallapiccola B Mereu ML Merlini L Pagliarani S. Low-rate repetitive nerve stimulation protocol in an Italian cohort of patients affected by recessive myotonia congenita. J Clin Neurophysiol. (2011) 28:39–44. 10.1097/WNP.0b013e31820510d7 21221019

[138] Suetterlin K Mannikko R Hanna MG. Muscle channelopathies: recent advances in genetics, pathophysiology and therapy. Curr Opin Neurol. (2014) 27:583–90. 10.1097/WCO.0000000000000127 25188014

[139] Park E MacKinnon R. Structure of the CLC-1 chloride channel from Homo sapiens. Elife. (2018) 7:e36629. 10.7554/eLife.36629 29809153 PMC6019066

[140] Wang K Preisler SS Zhang L Cui Y Missel JW Gronberg C. Structure of the human ClC-1 chloride channel. PLoS Biol. (2019) 17:e3000218. 10.1371/journal.pbio.3000218 31022181 PMC6483157

[141] Estevez R Schroeder BC Accardi A Jentsch TJ Pusch M. Conservation of chloride channel structure revealed by an inhibitor binding site in ClC-1. Neuron. (2003) 38:47–59. 10.1016/S0896-6273(03)00168-5 12691663

[142] Liantonio A Accardi A Carbonara G Fracchiolla G Loiodice F Tortorella P. Molecular requisites for drug binding to muscle CLC-1 and renal CLC-K channel revealed by the use of phenoxy-alkyl derivatives of 2-(p-chlorophenoxy)propionic acid. Mol Pharmacol. (2002) 62:265–71. 10.1124/mol.62.2.265 12130677

[143] Pusch M Accardi A Liantonio A Guida P Traverso S Camerino DC. Mechanisms of block of muscle type CLC chloride channels (Review). Mol Membr Biol. (2002) 19:285–92. 10.1080/09687680210166938 12512775

[144] De Boeck K Davies JC. Where are we with transformational therapies for patients with cystic fibrosis? Curr Opin Pharmacol. (2017) 34:70–5. 10.1016/j.coph.2017.09.005 28992608

